# Pancake kidney with bladder herniation

**DOI:** 10.1590/S1677-5538.IBJU.2014.0646

**Published:** 2015

**Authors:** Ihsan Yuce, Mecit Kantarci, Suat Eren, Akin Levent

**Affiliations:** 1 Ataturk University, School of Medicine, Department of Radiology, Erzurum, Turkey

## CASE PRESENTATION

**Figure 1:**
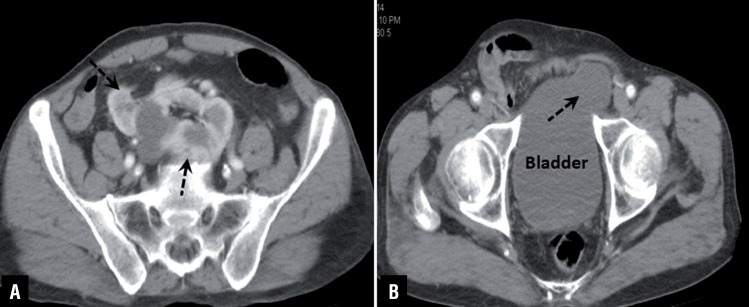
Axial CT images show pancake kidney and bladder herniation (dashed arrows).

**Figure 2:**
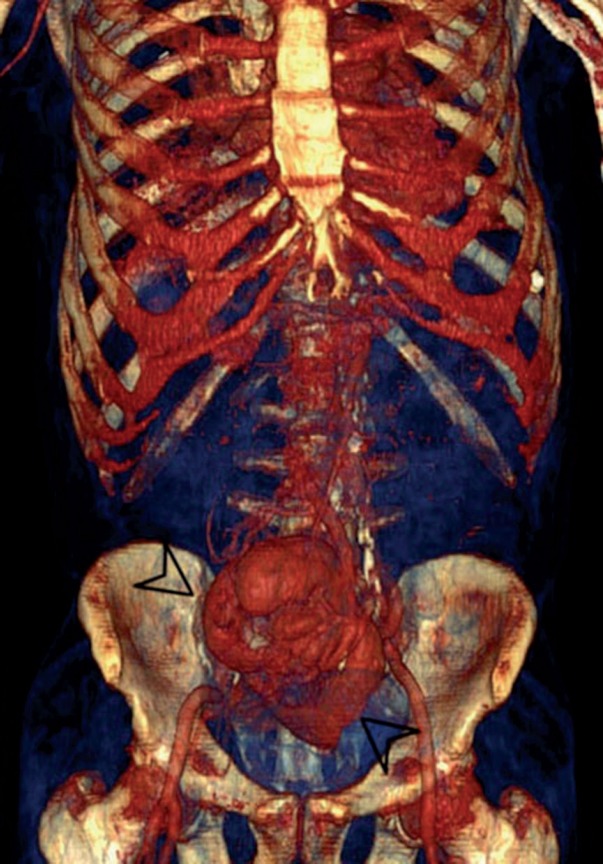
Three dimensional volume rendering image shows pancake kidney (arrowheads).

A 61-year-old man presented to the Emergency Department with vomiting and progressively worsening abdominal pain. A computed tomography (CT) was performed. The diagnosis of patient was acute cholecystitis and the patient was referred to general surgery clinic. In addition CT scan showed bilateral ectopic kidneys with urinary bladder herniation (Figures 1 and 2). Both kidneys were fused at the medial borders of each pole. To our knowledge, the case of pancake kidney with bladder herniation was not published yet in the literature.

Pancake kidney is very rare type of congenital fusion anomaly of the kidney. It is described as a renal mass located in the pelvis which is formed by complete medial fusion of renal parenchyma without intervening septum (1). Each kidney has its own collecting system and anteriorly placed short ureters entering the bladder orthotopically (1). The presence of a pancake kidney may predispose the formation of stones due to the probable rotation anomaly of the collecting system and short ureters which are prone to stasis and obstruction. Patients with pancake kidney are usually asymptomatic, but may present with features of urinary tract infection, fever and vague lower abdominal pain (1). If a pancake kidney has to undergo surgery, division of the parenchyma presents potential problems such as renal vascular damage, postoperative renal failure and eventual renal failure (2). Asymptomatic cases can be managed conservatively with long-term follow-up of renal function (1). If there are symptoms of renal failure, surgery is warranted. Ultrasonography, excretory urography and CT were efficient in detection and evaluation of pancake kidney anomaly (1).
